# Can virtual reality reduce anxiety and pain in dental patients?

**DOI:** 10.1038/s41432-025-01127-6

**Published:** 2025-02-20

**Authors:** Nicola Goodship, Greig Taylor

**Affiliations:** 1 General Professional Trainee, School of Dental Sciences, Newcastle University, Faculty of Medical Science, Newcastle, UK; 2 Clinical Lecturer in Paediatric Dentistry, School of Dental Sciences, Newcastle University, Faculty of Medical Science, Newcastle, UK

**Keywords:** Dentistry, Paediatric dentistry

## Abstract

**A Commentary on:**

**Nezhad H M, Ashourioun A, Sadeghdaghighi A**

The effect of virtual reality for anxiety and pain in dentistry: a systematic review and meta-analysis. *Community Dent Health* 2024; **41:** 248–255.

**Data sources:**

A systematic search was conducted across PubMed and Cochrane Library up to April 2024.

**Study selection:**

The article type was limited to Randomised Controlled Trials, comparing virtual reality (VR) interventions with non-VR methods in dental settings. The selection followed the PRISMA-P guidelines. Using the PICOS framework, studies involving dental patients of any age utilising VR during dental treatments and reporting outcomes on anxiety and pain were included.

**Data extraction and synthesis:**

Data extraction and quality appraisal were conducted independently by two reviewers using both the Grading of Recommendations, Assessment, Development, and Evaluations (GRADE) approach and the Risk of Bias 2 (ROB-2) tool. Meta-analyses used a random-effects model.

**Results:**

A total of 263 studies resulted in the inclusion of 27 studies, encompassing several dental treatments. Evidence quality ranged from low to moderate. Meta-analysis of 14 studies, encompassing 957 patients, revealed VR significantly reduced anxiety in children (SMD −1.44, 95% CI −2.24 to −0.63, low quality of evidence). Metal-analysis of five studies (485 patients) revealed VR had no effect on adults’ anxiety (SMD -0.35, 95% CI -1.11 to 0.4, low level of certainty). VR significantly reduced pain in both children (11 studies, 791 participants) (SMD −1.11, 95% CI −1.65 to −0.57, moderate level of certainty) and adults (6 studies, 557 participants) (SMD −0.59, 95% CI −1.187 to −0.001, low evidence quality). Heterogeneity was high across studies.

**Conclusion:**

The study concluded that VR is a promising intervention for reducing anxiety and pain in children during dental procedures, and pain reduction in adults. The authors suggested further research is needed to standardise the VR content and explore its impact across different age groups and dental procedures.

## GRADE Rating:



## Commentary

The fear of pain and dental anxiety are widely recognised as factors that can negatively impact the quality of care and may result in patients discontinuing treatment^1^. This avoidance of dental care contributes to poor oral health, including decay and tooth loss. Dentists often face challenges in effectively managing patients’ fear and anxiety^2^.

Several approaches have been used to manage dental pain and anxiety, including techniques such as guided imagery, hypnotherapy and acupuncture. Additionally, strategies such as enhancing patient control, voice control, behaviour shaping, the ‘tell-show-do’ technique, and distraction have also proven effective in alleviating anxiety and dental pain^3^.

One known distraction technique, virtual reality (VR), aims to immerse users in a three-dimensional environment, disconnecting them from their immediate surroundings^4,5^. Research on the use of VR in dental settings has been inconsistent and primarily focused on children. This quantitative systematic review and meta-analysis examined the impact of VR on reducing dental anxiety and pain across all age groups and dental conditions^6^.

The review was conducted with a strong methodological framework. It reports a clear aim and research question, employs a PICOS-guided search strategy, and presents a detailed PRISMA flow diagram^7^. This enhances the reliability of findings and allows the reader to interpret results with a high degree of confidence. The search encompassed two reputable primary databases, PubMed and the Cochrane Library. However, extending the search to include additional databases and grey literature could have further strengthened the review and reduced the risk of publication bias. Positively, the inclusion criteria included all patient groups, regardless of age, gender, or race, and included randomised controlled trials (RCTs) of various designs, including parallel, crossover, and split-mouth trials. Other study designs, for example cohort studies and case control studies, would not have been suitable to answer the research question and provide low quality evidence. The review was not limited by publication date. VR is likely to have advanced in recent years, and recent studies may not be comparable to older studies. A meta-analysis could have been completed grouping publication date on effect. Only studies published in English were included, and there is a risk that relevant studies published in other languages or using different terminologies were not identified.

Data extraction was performed by two independent researchers. The inclusion of a third reviewer could have helped resolve any disagreements and reduced the risk of subjective bias.

The 27 studies included in the review were conducted across various countries and published in reputable journals. Notably, 70% of the studies focused on the effects of VR on children and there was a ‘predominance of female gender’. This reduces generalisability of the review findings. Positively, the effects of VR were explored across a wide range of dental procedures, including the extraction of primary/permanent teeth, pulp therapy, restorative treatment and Inferior Dental Blocks. This increases external validity of the review.

A strength of this systematic review is the authors application of the GRADE assessment tool^8^, which provides guidance for how much confidence can be placed on findings. Additionally, two independent reviewers assessed bias using the Risk of Bias 2 tool^9^. The review authors are transparent in noting that all included studies had a high risk of bias, citing issues such as inadequate concealment of allocation sequences and deviations from the intended interventions. Notably, none of the studies employed double blinding, and 17 studies lacked pre-registered protocols. The quality of evidence was rated low to moderate. Appropriate statistical methods were used to assess heterogeneity, which was found to be high. Adopting a core-outcome set for dental pain and anxiety could support reduced heterogeneity in future reviews.

The high heterogeneity, low to moderate quality of evidence, and high risk of bias in the included studies limits the validity of results. The authors state that the results should be ‘interpreted with caution’.

This review provides valuable insights into the potential use of VR in dental settings, particularly for reducing dental anxiety and pain. It is important for researchers, especially those conducting RCTs, to minimise bias and control for confounding variables. Well-conducted research is needed to build upon these findings and strengthen the evidence base for VR use in dentistry.

Practice points

- **Use of VR**: VR shows promising potential in reducing dental anxiety and pain, suggesting it could be a valuable tool in dental care.
- **Ongoing Research**: Encourage further research exploring the use of VR in a dental setting. It is important for researchers, especially those conducting RCTs, to minimise bias.
- **Use of Validated Tools**: Researchers should prioritise using validated tools that align with the objectives of their studies to ensure reliability and consistency in results.
- **Standardised VR Protocols**: A standardised VR protocol for both children and adults should be developed to ensure its consistent application across different dental practices.
